# Effects of Sugarcane-Derived Polyphenol Supplementation on Methane Production and Rumen Microbial Diversity of Second-Cross Lambs

**DOI:** 10.3390/ani14060905

**Published:** 2024-03-14

**Authors:** Pragna Prathap, Surinder S. Chauhan, Matthew Flavel, Shane Mitchell, Jeremy J. Cottrell, Brian J. Leury, Frank R. Dunshea

**Affiliations:** 1 School of Agriculture, Food and Ecosystems Sciences, Faculty of Science, The University of Melbourne, Parkville, Melbourne, VIC 3010, Australia; ss.chauhan@unimelb.edu.au (S.S.C.); jcottrell@unimelb.edu.au (J.J.C.); brianjl@unimelb.edu.au (B.J.L.); 2 TPM Bioactives Division, The Product Makers Pty Ltd., Keysborough, Melbourne, VIC 3173, Australia; mflavel@tpm.com.au (M.F.); smitchell@tpm.com.au (S.M.); 3 Department of Microbiology, Anatomy and Physiology, La Trobe University, Bundoora, Melbourne, VIC 3086, Australia; 4 Faculty of Biological Sciences, The University of Leeds, Leeds LS2 9JT, UK

**Keywords:** methane, microbiota, polyphenols, sheep

## Abstract

**Simple Summary:**

Enteric methane emission reduction from livestock is one of the most discussed topics of the 21st century. Plant-based polyphenols are found to be one of the effective solutions to reduce methane emissions from ruminant animals. Hence, this study evaluates the effect of sugarcane-based polyphenolic supplements on enteric methane emission and its effect on microbiota and body weight changes in sheep. The results from this study indicate that both dosages of Polygain reduced methane emission from sheep and increased average daily gain compared to the control group animals with notable changes in rumen microbiota.

**Abstract:**

The objective of this study was to evaluate the effects of feeding sugarcane-derived polyphenolic supplement (Polygain, The Product Makers Australia, Keysborough, VIC, Australia) on enteric methane (CH_4_) emission, rumen microbiota, and performance of second-cross lambs. For this purpose, 24 Poll Dorset × (Border Leicester × Merino) lambs were allocated to 3 different treatments: Control (C), 0.25% Polygain (0.25 PG), and 1% Polygain (1 PG) diets with a uniform basal feed (25% cracked wheat grain, 25% cracked barley grain, 25% oaten chaff, 25% lucerne chaff). Both doses of Polygain reduced the total CH_4_ production (g/day; *p* = 0.006), CH_4_ yield (CH_4_, g/kg of dry matter intake; *p* = 0.003) and CH_4_ intensity (CH_4_, g/kg of BW; *p* = 0.003). Dry matter intake tended to be greater (*p* = 0.08) in sheep fed 1 PG compared to the C group, with the 0.25 PG group being intermediate. The average daily gain of the lambs was improved (*p* = 0.03) with 1% Polygain supplementation. The relative abundance of genera *Methanobrevibacter_*unidentified, *Methanomethylophilaceae_*uncultured, *Methanogenic archaeon* mixed culture ISO4-G1, *Methanosphaera* uncultured rumen methanogen, *Methanogenic archaeon* ISO4-H5, and *Methanobrevibacter boviskoreani* JH1 were reduced with Polygain supplementation. In conclusion, feeding Polygain reduced lambs’ enteric CH_4_ emissions, altered the rumen microbiome, and improved the growth performance of lambs.

## 1. Introduction

Climate change is one of the major concerns of the present era with the accumulation of atmospheric greenhouse gases (GHG) being one of the major causes. Compared with the 2021 level, global GHG emissions have increased by around 1.2% in 2022 and the current estimate of emissions is 57.4 gigatons of CO_2_ equivalent [1,2]. Methane (CH_4_) is one of six GHGs that must be reduced under the Kyoto Protocol agreement, and the agriculture sector is responsible for the most CH_4_ emissions [3]. The CH_4_ emission from the livestock sector accounts for 32% of anthropogenic CH_4_ emissions [4]. As CH_4_ has 80 times the global warming potential of CO_2_ and shorter shelf life, cutting CH_4_ emissions can reduce the greenhouse effect and global warming faster than focusing on CO_2_ alone [5]. The major challenges in mitigating CH_4_ emissions from the livestock sector include complex and diverse livestock production systems, increasing costs and demand for livestock products, and less availability and adoption of emission reduction strategies.

There is a growing need to identify natural feed additives that can reduce CH_4_ emissions. Recently, plant herbal extracts have gained popularity among farmers and researchers for their potential to reduce methane emissions, improve nitrogen metabolism, and replace antibiotics [6]. As a result, several studies have investigated the effect of dietary additions of plant extracts, plant secondary metabolites, and essential oils on enteric CH_4_ emissions [7,8,9]. Several plant-based supplements have been found useful in reducing enteric CH_4_ emissions from ruminants because of their chemical properties and ability to alter the microbiota to produce less CH_4_ [10,11]. A major class of plant-derived chemicals implicated in contributing to the reduction of methane emissions include polyphenols [12]. Polyphenols are plant secondary metabolites that contain a phenolic group, characterised by the presence of at least one hydroxyl group as a substituent. Polyphenols can be either simple, such as gallic acids and ellagic acids, or they can be dimeric, oligomeric, or polymeric compounds [13,14]. Polygain is a natural sugarcane extract that contains simple polyphenols such as derivatives of gallic acid. It has shown positive effects on various animal species, including body weight gain, meat quality, and physiological variables in heat-stressed broiler chickens [15]. Additionally, it has been associated with methane reduction and improvements in milk production in dairy cows [16]. The present study aimed to determine the effects of supplementing different dosages of sugarcane-derived polyphenols on enteric CH_4_ production, dry matter intake (DMI), average daily gain (ADG), feed conversion efficiency (FCE), and rumen microbial profile.

## 2. Materials and Methods

All procedures involving animals were approved by the animal ethics committee of the Faculty of Veterinary and Agriculture Sciences (FVAS), the University of Melbourne (2287 Version 2.4).

### 2.1. Animals, Housing, Diets

Twenty-four second-cross Poll Dorset × (Border Leicester × Merino) lambs of mean body weight 38.7 ± 1.4 kg were used in this experiment. Lambs were randomly allocated to one of the dietary treatment groups, Control (C), 0.25% Polygain (0.25 PG), or 1% Polygain (1 PG). The feed was offered at 2 × maintenance level intake [17], and the basal (Control) diet contained 25% crushed wheat, 25% barley, 25% oaten chaff, and 25% lucerne chaff (Table 1) on a DM basis. The feed analysis was performed in accordance with the Australian Fodder Industry Association Laboratory Method with the help of DPI Laboratory services, NSW, Australia. Polygain™ (The Product Makers Australia, Keysborough, VIC, Australia), which is a commercially available natural polyphenol extract derived from sugarcane, was mixed with the basal diet in a portable cement mixer at either 0.25 or 1%. The daily feeding ration was split into two: morning feeding at 09:00 h and afternoon feeding at 13:00 h. Initially, animals were acclimatized to indoor housing conditions in group pens for 5 days. Afterwards, they were moved to individual pens with sawdust on the concrete floor and adapted to the feed additive and grain diets for 15 days. The subsequent measurement period lasted for 16 days and the sheep had ad libitum access to fresh water. At the end of the experimental period, animals were sacrificed to obtain rumen fluid for the 16S rRNA sequencing. Body weight was measured every 8 days using a walk-over scale before morning feeding. The orts were collected and weighed every morning before feeding to calculate DMI.

### 2.2. Enteric Methane Measurement

**Figure 1:**
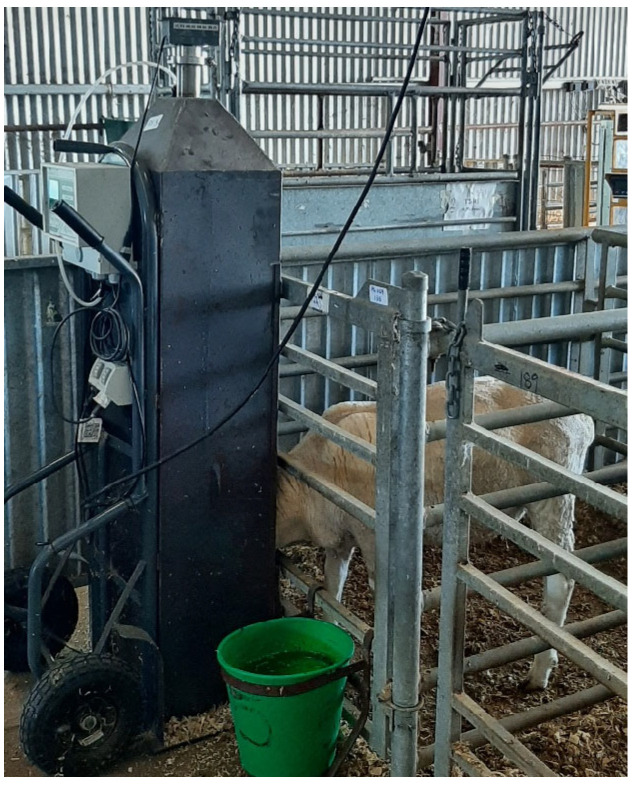
Instrumental setup for the enteric methane estimation from the sheep.

The enteric CH_4_ was collected across the experiment using a hooded infrared CH_4_ analyser (Guardian NG gas card, Edinburgh Instruments Ltd., Livingston, UK) attached to the feed bins (Figure 1) [18]. The sensors were calibrated each morning with 1% CH_4_ and 0% CH_4_ gas (Noventis Australia Pty Ltd., Melbourne, VIC, Australia). The CH_4_ analysers were equipped with sensors and a datalogger that can measure and accrue CH_4_ data every 5 s. The equipment had an enclosure on 3 sides to prevent air mixing and skewing of CH_4_ readings. Sheep were trained to eat feed from bins with an enclosed hood over the 15-day acclimation period. Nylon tubes were used for gas collection to reduce memory effects and eliminate false readings [19].

### 2.3. Rumen Fluid Sampling and DNA Extraction, Library Preparation, and Bioinformatics

At the completion of this study, the sheep were commercially slaughtered in a mobile butchery and, within 10 min of exsanguination, representative samples were collected from 4 corners of the rumen. The samples were flash frozen with liquid nitrogen and stored at −80 °C until analysis. Later, the digesta samples were thawed, and gDNA was isolated using the QIAamp^®^ Fast DNA Stool Mini kit (Qiagen, Hilden, Germany) with some modifications, as suggested by Knudsen, et al. [20]. The extracted gDNA was then sent to the Australian Genome Research Facility, Victoria, Australia, for sequencing and PCR amplification. V3 and V4 regions of 16S rRNA were performed using PCR with CCTAYGGGRBGCASCAG as a forward primer (341F) and GGACTACNNGGGTATCTAAT as a reverse primer (806R). Thermocycling was performed with an Applied Biosystem 384 Veriti and using Platinum SuperFi II master mix (Invitrogen, Parkville, VIC, Australia) for the primary PCR. Magnetic beads were used for cleaning the first stage PCR, and samples were visualised on 2% Sybr Egel (Thermo-Fisher, Waltham, MA, USA). Using the same polymerase master mix, a secondary PCR was performed to index the amplicons. Amplicons were then cleaned again using magnetic beads, quantified using fluorometry (Promega Quantifluor, Madison, WI, USA), and normalised. For the final time, the equimolar pool was cleaned, magnetic beads were used to concentrate the pool, and measurement was carried out using High-sensitivity D1000 Tape on an Agilent 4200 TapeStation (Agilent Technologies, Santa Clara, CA, USA). The pool was then diluted to 5 nM and the molarity was confirmed again using a Qubit high-sensitive dsDNA assay (Thermo-Fisher). DNA was then sequenced on an Illumina MiSeq (San Diego, CA, USA) using the V3, 600 cycle kit (2 × 300 base pairs paired end). Paired-end reAd mergeRPEAR (PEAR Version 0.9.5) software was used to assemble and merge the paired-end reads by aligning forward and reverse reads [21]. The primers were identified and trimmed, and then processed with the help of Quantitative Insights into Microbial Ecology (QIIME 1.8) [22] USEARCH Ver. 7.1.1090 [22,23] and UPARSE software [24]. The Usearch sequence analysis tool was used to filter sequences by quality, remove full-length duplicate sequences, and sort data by abundance. Singletons or unique reads were discarded from the data set. Based on the “rdp_gold” database as a reference, sequences were clustered and then chimera filtered. Reads were mapped back to OTUs with a minimum identity of 97% to determine the number of reads in each out. The QIIME taxonomy was assigned using the Greengenes database (version 13.8, August 2013) [25].

### 2.4. Statistical Analysis

All the statistical analyses were performed using Genstat 16th edition (Version 16.1.0.10916, VSN International Ltd., Hertfordshire, UK). Restricted Maximum Likelihood analysis (REML) was used to test for significant differences among the treatments. Treatment was considered as the fixed effect while replication and animal were considered random variables. A *p*-value of ≤0.05 was considered significant, and a *p*-value between 0.05 and 0.1 was considered a trend.

## 3. Results

Overall, Polygain treatment resulted in a reduction of enteric CH_4_ emissions from the second-cross lambs (Table 2). The total CH_4_ production (CH_4_ g/day) was lower for 0.25 PG than the higher dosage of 1 PG, which, in turn, was lower than the control group (*p* = 0.006). When expressed in terms of DMI, the CH_4_ yield was reduced (*p* = 0.003) by 52% and 37% in the 0.25 PG and 1 PG groups, respectively. Similarly, emission intensity (CH_4_, g/kg of BW) displayed a similar trend with a 51% reduction from the 0.25 PG group and a 36% reduction from 1 PG lambs (*p* = 0.003).

While there was no significant effect of Polygain feeding on the DMI, lambs consuming 1 PG tended to have higher DMI than control lambs (*p* = 0.08; Table 2). The 1 PG group of lambs had higher ADG (*p* = 0.03) and FCR (*p* = 0.04) than the 0.25 PG and control group animals (Table 2).

**Figure 2:**
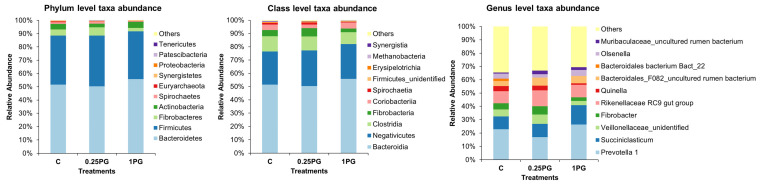
Effect of different concentrations of Polygain on the rumen microbial composition of second-cross lambs. Treatments: C—control group, 0.25 PG—0.25% Polygain supplemented group, 1 PG—1% Polygain supplemented group.

In general, a total of 19 eukaryotic phyla, 32 classes, and 254 genera were identified in the rumen fluid of second-cross lambs (Figure 2). Among the 10 major abundant phyla identified, *Bacteroidetes* (52.6 ± 0.03%), *Firmicutes* (37.0 ± 0.01%), *Fibrobacteres* (4.6 ± 0.02%), and *Actinobacteria* (3.8 ± 0.01%) were more abundant, and *Patescibacteria* (0.1 ± 0.00%) and *Tenericutes* (0.1 ± 0.00%) were the least identified. At the class level, the rumen fluid had a higher abundance of *Bacteroidia* (52.6 ± 2.9%), *Negativicutes* (26.0 ± 1.1%), *Clostridia* (10.3 ± 1.3%), and a lower abundance of *Methanobacteria* (0.3 ± 0.2%) and *Synergistia* (0.2 ± 0.1%). Further, *Prevotella* (22.1 ± 4.7%) and *Succiniclasticum* (11.3 ± 2.7%) were two major prevailing genera in the rumen fluid of second-cross lambs. Irrespective of the dosage, dietary supplementation with Polygain reduced the number of *Euryarchaeota*/*Methanobacteria*. Within the class *Methanobacteria*, genera *Methanobrevibacter* and *Methanosphaera* were higher in the control diet than in the 0.25 PG group of lambs, which in turn was higher than the 1 PG group. Further, the familiae *Ruminococcaceae*, *Lachnospiraceae*, and *Christensenellaceae,* which are associated with higher CH_4_ emissions, were more prevalent in the rumen fluid of control group lambs than in the 1 PG and 0.25 PG lambs.

## 4. Discussion

The major finding from the present study was that dietary supplementation of Polygain significantly reduced enteric CH_4_. This reduction was achieved with positive effects on productive performance. These data also demonstrated that both 1 PG and 0.25 PG substantially altered the rumen microbiome profile with prominent reductions in the methanogenic community. The extract from Australian sugarcane, Polygain, contains polyphenols, flavonoids other plant secondary metabolites [26].

While the CH_4_ reduction was maximised at 0.25 PG level, there appeared to be a linear effect on ADG and DMI within the dose range investigated (up to 1% inclusion). The amount of feed offered was dependent upon body weight so to some extent the increase in ADG at least partially drove the DMI response. Also, the taste of sugarcane may have stimulated feed intake [27]. The increase in the ADG in our study could be ascribed to the presence of flavonoids in the Polygain, as plant flavonoids have been shown to improve growth performance, digestion, immune function, and reproductive functions in animals [28,29]. A similar result of improved weight gain was found in MeHgCl intoxicated rats supplemented with sugarcane juice [30]. In agreement with our results, Shakeri, et al. [15] also observed positive effects of Polygain supplementation on ADG and FCE in heat stressed and thermoneutral Ross-308 chicks. The polyphenols present may bind some of the protein and increase the amount of rumen undegradable digestible protein (RUDP), which can increase ADG under some circumstances and decrease CH_4_ emissions. In this context, Lamba, et al. [31] found that increasing RUDP was associated with decreased in vitro CH_4_ production, which supports this concept. However, these results would need to be confirmed in studies with a longer period of feeding as the results observed in the current study are of relatively short duration.

Sugarcane-derived polyphenol supplementation decreased CH_4_ emission from sheep by 49% and 33% for 0.25 PG and 1 PG doses, respectively. Flavonoids and polyphenols present in the Polygain could be the reason behind the decline in the CH_4_ g/day as they possess anti-methanogenic and antiprotozoal effects [32,33]. Similar to our results, Ahmed, et al. [16] observed a reduction in CH_4_ from dairy cows supplemented with 0.25% Polygain. Further, Mao, et al. [34], Cieslak, et al. [35], and Chen, et al. [36] observed a reduction in enteric CH_4_ production in Huzhou lambs and Polish Holstein–Friesian dairy cows and Dorper × small-tailed Han ewes, respectively, with the supplementation of plant-derived bioactive compounds such as Mulberry leaf flavonoid and Resveratrol and they have partially attributed this reduction to anti-microbial and protozoal effects of phenolic and flavonoid compounds in the phytoextracts. Considering the available literature and product information, we speculate that Polygain has CH_4_ mitigation potential due to its ability to target methanogenic archaeal populations and enrich bacteria that produce less hydrogen.

Phylum *Euryarchaeota* members primarily use hydrogen, an end product of rumen fermentation, to reduce CO_2_ and to form CH_4_ [37]. Irrespective of the dosage, polyphenols present in the Polygain are shown to reduce the CH_4_ from the second-cross lambs and these are supported by the reductions in the *Methanobrevibacter*, *Methanomethylophilaceae_uncultured*, *Candidatus Methanomethylophilus* and *Methanosphaera* populations. Similar results of polyphenol-induced reductions in methanogens and corresponding CH_4_ reductions were also observed in in vitro [38] studies and in vivo studies [39]. Even though the 1 PG group had lower methanogen abundance than the 0.25 PG group, the measure of enteric CH_4_ output in the 0.25 PG group was lower than in the 1 PG group, suggesting that archaeal community in 0.25 PG animals may have a lower CH_4_-emitting activity than their protozoa counterparts [40,41].

## 5. Conclusions

The addition of Polygain to sheep diets decreased enteric CH_4_ production and intensity improved short-term productive performance. Supplementation of Polygain reduced enteric CH_4_, presumably by acting as an anti-methanogenic agent. Among the two different dosages (0.25 PG and 1 PG), the lowest dosage of 0.25 PG could be recommended for reducing enteric CH_4_ emissions from ruminant animals. However, there does appear to be growth responses beyond this dose up until at least a 1% inclusion rate.

## Figures and Tables

**Table 1:** Feed components and composition of the control diet.

	Control Diet
Feed components, %	
Crushed Wheat	25.0
Crushed Barley	25.0
Oat Chaff	25.0
Lucerne Chaff	25.0
Analysed variables	
DM Digestibility, %	80.8
Digestible Organic Matter, %	77.8
Metabolizable Energy, MJ/kg	12.1
Crude Protein, %	13.4
Ether Extract, %	1.95
Starch Total, %	40.8
Ash, %	4.75
Organic Matter, %	95.3
Neutral Detergent Fibre, %	31.8
Acid Detergent Fibre, %	15.5

**Table 2:** Effect of feeding different dosages of Polygain on enteric methane emission, dry matter intake, average daily gain, feed conversion efficiency of second-cross lambs.

Parameters	Treatments			SED	*p*-Values
	C	0.25 PG	1 PG		
Total methane production (CH_4_, g/day)	27.0 ^a^	13.7 ^b^	18.0 ^b^	3.69	0.006
Methane yield (CH_4_, g/kg of DMI)	22.6 ^a^	10.9 ^b^	14.3 ^b^	3.08	0.003
Emission intensity (CH_4_, g/kg of/BW)	0.70 ^a^	0.34 ^b^	0.45 ^b^	0.09	0.003
Dry matter intake (kg/day)	1.18 ^a^	1.22 ^a^	1.25 ^a^	0.03	0.083
Average daily gain (g/day)	2.40 ^a^	67.3 ^a,b^	135.5 ^b^	47.0	0.034
Feed conversion efficiency (g/g)	0.00 ^a^	0.06 ^a,b^	0.11 ^b^	0.04	0.042

CH_4_—methane, DMI—dry matter intake, BW—body weight, SED—standard error of differences, C—control group, 0.25 PG—0.25% Polygain supplemented group, 1 PG—1% Polygain supplemented group. Data accompanied by distinct superscript alphabets indicate significant dissimilarities between groups.

## Data Availability

Data are available on request due to restrictions.
